# Prevalence of cryptococcal meningitis among people living with human immuno-deficiency virus and predictors of mortality in adults on induction therapy in Africa: A systematic review and meta-analysis

**DOI:** 10.3389/fmed.2022.989265

**Published:** 2022-09-08

**Authors:** Seke G. Y. Muzazu, Dawit Getachew Assefa, Christabel Phiri, Tewodros Getinet, Samrawit Solomon, Gizachew Yismaw, Tsegahun Manyazewal

**Affiliations:** ^1^Centre for Innovative Drug Development and Therapeutic Trials for Africa (CDT-Africa), College of Health Sciences, Addis Ababa University, Addis Ababa, Ethiopia; ^2^Enteric Diseases and Vaccines Research Unit, Centre for Infectious Disease Research in Zambia (CIDRZ), Lusaka, Zambia; ^3^Department of Nursing, College of Health Science and Medicine, Dilla University, Dilla, Ethiopia; ^4^Levy Mwanawasa University Teaching Hospital, Department of Internal Medicine, Lusaka, Zambia; ^5^School of Public Health, Saint Paul’s Hospital Millennium Medical College, Addis Ababa, Ethiopia

**Keywords:** HIV, cryptococcal meningitis, antifungal therapy, amphotericin B, flucytosine, fluconazole, systematic review, meta-analysis

## Abstract

**Background:**

Cryptococcal meningitis (CM) is a leading cause of adult meningitis in countries with a high burden of HIV. It has remained a significant cause of morbidity and mortality in Africa despite the extensive rollout of HIV antiretroviral therapy (ART). This study aimed to systematically synthesize the evidence on the prevalence of CM among people living with HIV (PLWH) and its predictors of mortality among adults who are on induction antifungal therapy in Africa.

**Methods:**

PubMed/MEDLINE, Embase, and Google Scholar were searched for randomized clinical trials or observational studies published in Africa from 1995 to April 2021. Pooled prevalence of CM among PLWH was calculated using R-studio Version 1.4.1717 software and the data extracted from eligible studies were pooled as percentage with a 95% confidence interval (CI). Predictors of mortality among adults on induction antifungal therapy were synthesized narratively.

**Results:**

Out of 364 studies identified, 17 eligible articles were included in the analysis. The prevalence of CM among PLWH in Africa was 5.11% (95% CI 2.71–9.43%; participants = 10,813; studies = 9; *I*^2^ = 97%). In the subgroup analysis, the prevalence was 12.9% (95% CI 4.883–30.0; participants = 533; studies = 3; *I*^2^ = 63%) in the years 1995–2010 and 3.18% (95% CI 1.54–6.45; participants = 10,280; studies = 6; *I*^2^ = 98%) in the years 2011–2021, with the prevalence significantly decreased by 51% (*p* = *0.02*). Predictors of mortality were fluconazole monotherapy, focal neurological signs, low Glasgow coma scale, and delayed diagnosis of CM at varied timepoint.

**Conclusion:**

Prevalence of CM has significantly decreased from 1996–2010 to 2011–2021 among PLWH on induction therapy in Africa. Fluconazole monotherapy, focal neurological symptoms, diastolic blood pressure < 60 mmHg, and concurrent tuberculosis coinfection were significant predictors of mortality at 2- and 10-weeks timepoints. CM remains a major concern among PLWH despite increases in ART coverage. Improved access to effective antifungal therapies is needed in Africa for timely initiation of combination induction therapy and better treatment outcomes of PLWH.

**Systematic review registration:**

[https://www.crd.york.ac.uk/prospero/display_record.php?RecordID=254113], identifier [CRD42021254113].

## Introduction

Cryptococcal Meningitis (CM) is one of the top causes of meningitis in adults in Sub-Saharan Africa (SSA) and other regions with a high prevalence of Human Immuno-deficiency Virus (HIV), accounting for over 100,000 incident cases of meningitis per year in the region. Globally, about 15% of HIV-related deaths are attributable to CM and 75% of these occur in SSA (1–3). It was further estimated that 10–20% of HIV-related deaths in Africa are due to CM (4). Although the global incidence and deaths from HIV have continued to decline owing to increased access to antiretroviral therapy (ART), including pre- and post-exposure prophylaxis, the prevalence of the disease has not declined (5).

CM, an immune-compromise-associated fungal infection caused by *Cryptococcus neoformans* or *Cryptococcus gattii*, has a high mortality rate in both HIV-negative and HIV-positive individuals, with a cumulative mortality of about 65% at 1 year (4, 6, 7). It is commonly diagnosed in HIV-positive patients with a CD4 count lower than 100 cells/μl. Mortality from the disease has remained high despite increased ART access and this is true for 20–50% of hospitalized patients (6, 8, 9) and commonly in the immuno-compromised (7, 10) and those with a history of defaulting or other comorbid conditions (11).

Adequate antifungal therapy and physical removal of excess cerebrospinal fluid (CSF) to relieve acute symptoms are the mainstays of CM treatment (12, 13). The World Health Organization 2018 guidelines targeting low- and middle-income countries recommend the use of amphotericin B (AmB) dosed at 1 mg/kg/day for a week with flucytosine (5-FC) in four divided doses of 100 mg/kg/day. To complete the induction therapy phase, fluconazole (FLU) at 1200 mg/day is also administered in the following week, and 800 mg/day of FLU for 8 weeks and 200 mg/day are given in the consolidation and maintenance phases, respectively (14). Though preferable, AmB has not been widely available across the SSA due to high cost, thereby making FLU monotherapy the mainstay of therapy (15, 16). Depending on availability, drugs recommended for the induction phase included a 2 weeks FLU and 5-FC or a 2 weeks AmB and FLU (14). Though AmB is one of the most effective, it has been associated with nephrotoxicity and anemia which have limited its use in patients with preexisting renal dysfunction or baseline hemoglobin level of < 8 g/dl (12, 17, 18).

Several studies have documented a significant association between early mortality and altered level of consciousness or sensorium. Patients who presented with either a low Glasgow coma scale, altered behavior, or simply altered mental status died or had a longer hospital admission (19–22). Furthermore, a CD4 count between 100 and 200 cells/mm^3^ predisposes one with CM to worse symptoms and clinical outcomes (23). Anemia is a hematological finding that has been reported as a significant contributor to 2 and 10-week mortality if present in the moderate to severe forms at baseline (24).

Survival of HIV-related CM patients has improved in the advent of ART compared to previous periods, while mortality in HIV-related CM has remained high (20, 25, 26). Individual studies on the subject of mortality predictors in HIV-related CM either used low sample sizes, compared only baseline variables at study entry points, considered patients not naïve to therapy, or focused on a single antifungal regimen (27, 28). Further, some studies have stated that CM is a metric of HIV treatment failure and so it becomes important to consider all the factors contributing to CM mortality to better assess HIV programming and make informed policy recommendations (3). Thus, it is important to understand the prevalence of CM among people living with HIV (PLWH) and predictors of mortality in patients on induction therapy in high HIV-endemic settings.

To the best of our knowledge, few reviews have been done documenting the pooled continental prevalence of the disease in Africa or short to medium-term mortality determinants of HIV-related CM among adults for the region (29–31). This review aimed to synthesize pooled prevalence of HIV-related CM and identify predictors of mortality among patients on induction antifungal therapy for the disease.

## Methods

The study protocol was registered on the International Prospective Register of Systematic Reviews (PROSPERO) database, ID: CRD42021254113. The methods and findings of the review have been reported according to preferred reporting for systematic reviews and meta-analyses (PRISMA 2020) (32).

### Eligibility

Eligible studies were identified using the population, intervention, comparison, outcome and study design (PICOS) model.

• **Participants**
  – Adult male and non-pregnant female with confirmed HIV and CM.
• **Intervention**
  – Anti-CM therapy, which could be AmB based regimens, 5-FC, or FLU, or a combination of either of these drugs.
• **Outcomes**
  – Primary outcome:
    ○ The prevalence of HIV-related CM in Africa. Prevalence data on CM was obtained by calculating the number of PLWH for whom CM was confirmed amongst the total number of PLWH included during the data collection period.
  – Secondary outcomes:
    ○ Pooled prevalence of cryptococcal antigenemia;
    ○ All-cause mortality reported by 2 weeks (for short-term) and by 10-weeks (for medium-term) of therapy, and their association with identifiable predictors of mortality;
    ○ Predictors of mortality, including a history of or persisting headache, nausea and vomiting, visual disturbance, history of tuberculosis, and history of ART use.
    ○ Physical examination predictors include fever, Glasgow coma scale of less than 15/15, neck stiffness, altered mental status, and high opening pressure (> 25 mmH_2_O).
    ○ Other predictors including aneamia (heamo globin < 7.5 g/dl), low CD4 (200 copies/ml), high CSF white cell count (> 5 cells/mm^3^), low plasma glucose (< 0.6 mmol/l), high CSF protein (> 0.5 mg/dl), high fungal burden and evidence of renal dysfunction.
• **Studies**
  – Randomized controlled trials and observational studies published from 1995 to April 2021 and written or translated into English.

### Electronic searches

A systematic literature search was done to identify relevant articles from online databases PubMed/MEDLINE, Embase, and Google Scholar. Studies were retrieved using medical subject headings (MeSH) and a combination of subject words and free words. The search term for PubMed was (((“meningitis, cryptococcal”[MeSH Terms] OR “cryptococcal”[Text Word] OR “cryptococcosis”[Text Word] OR “Cryptococcus neoformans”[Text Word] OR “cryptococcus gattii”[Text Word] OR “cryptococc*”[Text Word]) AND (“HIV”[MeSH Terms] OR “human immunodeficiency virus”[Text Word] OR “AIDS”[Text Word])) AND (“Death”[MeSH Terms] OR “Mortality”[MeSH Terms] OR “Mortality”[MeSH Subheading] OR “death*”[Text Word] OR “died”[Text Word] OR “non-survivor*”[Text Word] OR “non-survival”[All Fields] OR “poor outcome*”[Text Word] OR “fatal”[Text Word])) AND (“Africa”[MeSH Terms] OR Africa[Text Word]). A detailed search strategy for all databases is found in the Supplementary Appendix 1. Selected study references were checked for possible additional papers.

### Study selection

Two independent reviewers (SGYM, DGA) performed the search selected articles by title and abstract and non-relevant articles were excluded. From the title and abstract of all publications, those that were duplicated or did not meet the inclusion criteria were excluded. The full articles of the remaining studies were further reviewed. Any disagreements between reviewers were solved through consensus.

### Data extraction

The identified data were listed, and information was provided based on the study’s outcomes of interest. For the study on the primary outcome, thus the prevalence of CM, the data extraction format included the surname of the first author, year and country of publication, sample size, the total number of cases, and prevalence of CM with 95% CI. For studies on the secondary outcomes, the data extraction format included the surname of the first author, year and country of publication, study period, study design, sample size, demographic, and baseline characteristics of the participants, ART history, clinical and laboratory features at presentation and during admission, interventions, follow-up period, and outcomes.

### Quality and risk of bias assessments

We used the Newcastle Ottawa Scale (NOS) for assessing the quality of included observational studies (33). This ROB tool can be used for cohort and cross-sectional studies, and it includes three main domains and eight subdomains covering selection, comparability, and outcome. Each subdomain receives 1 star with 2 stars being maximum for others and scores range from 0 to 9. The risk of bias for each trial was evaluated by two review authors independently using the Cochrane Collaboration’s tool for assessing the “Risk of bias.” This guidance was used to decrease the risk of bias amongst six domains: sequence generation; allocation concealment; blinding (of participants, personnel, and outcome assessors); incomplete outcome data; selective outcome reporting; and other sources of bias. The risks were classified as high risk, unclear risk, and low risk (34). To assess the possibility of publication bias, a funnel plot for asymmetry (Egger’s test *P* < 0.05) was used.

### Data management and statistical analysis

Selected studies were imported from databases using ENDNOTE X7 software. Data extracted from selected studies was first summarized in Microsoft Excel. To make the distribution of the data normal, we first transformed the data using the logit transformation. Meta-analysis was done using R-studio Version 1.4.1717 software to determine the pooled prevalence of HIV-related CM. We also conducted a trend of prevalence comparison for the period before and after 2010 taking into account the IDSA updated guideline on cryptococcal meningitis management which included PLWH as a risk group (35). A systematic narrative synthesis was done for mortality predictors and results were summarized using texts and tables. Studies were shown in forest plots in chronological order of the year the studies were published. A random-effects model was used since trials were done by different researchers operating independently, and it could be implausible that all trials had functional equivalence, with a common effect estimate.

Heterogeneity among the included studies was assessed by inspecting the forest plots and the Cochrane Q and *I*^2^ statistic used to measure heterogeneity among the trials in each analysis, the Chi^2^ test with a *P* < *0.10* to indicate statistical significance was used. The results were interpreted following Cochrane Handbook for Systematic Reviews of Interventions Version 6.0 (36). Subgroup analysis was done by dividing the studies into two groups based on the year when the studies were conducted. Meta-regression was used to investigate the association of study characteristics that cause heterogeneity with the prevalence. The covariates were Year the study was conducted. To assess the influence of small-study effects on the results of our meta-analysis, fixed-effect and random-effects estimates of the intervention effect were compared.

### Statement of ethics compliance

This article was based on previously conducted studies and did not contain any studies with human participants or animals performed by any of the authors.

## Results

### Search results

**FIGURE 1:**
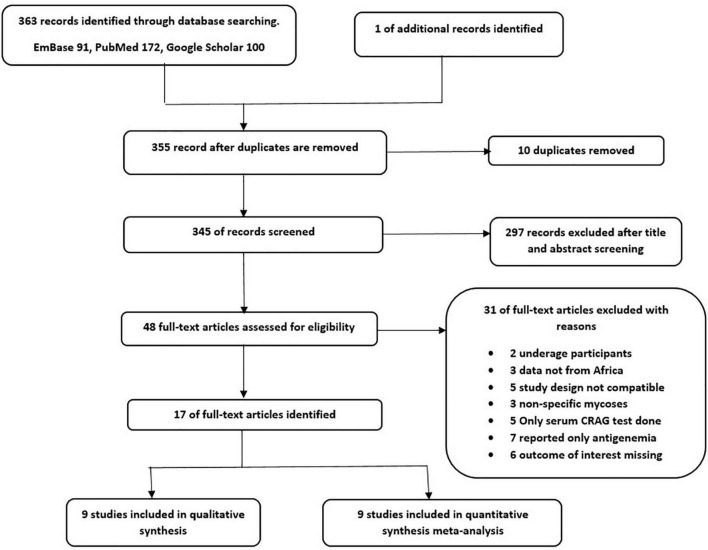
PRISMA Flow diagram of literature selection (1 study was used in both narrative synthesis and meta-analysis).

After a systematic searching of databases and other sources, 364 studies were retrieved and sequentially screened for eligibility, after which 17 studies were included. Figure 1 summarizes the PRISMA flowchart of the study.

### Study characteristics

A total of 17 studies were included in this review with 9 being used for the meta-analysis and 9 for the qualitative/narrative synthesis. One study (37) reporting both prevalence and mortality predictors was included in both the narrative review and meta-analysis.

**FIGURE 2:**
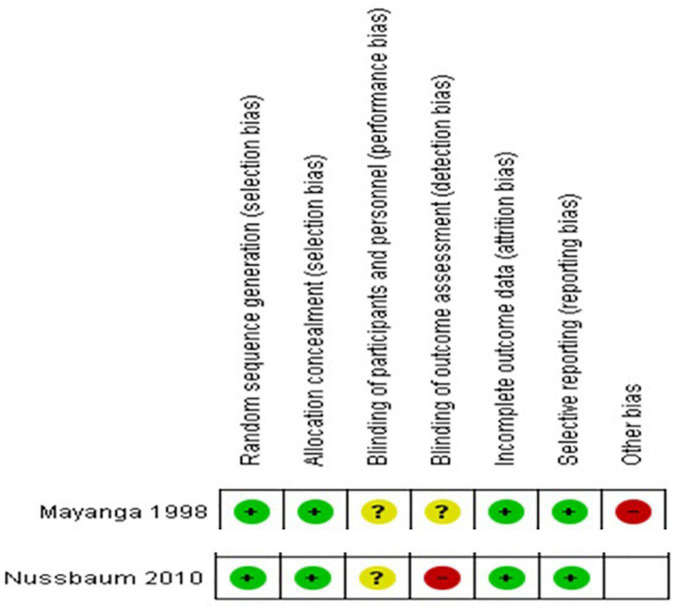
Risk of bias summary- review authors’ *judgments* about each risk of bias item for each included RCT.

In our NOS risk of bias assessment, all our studies scored from 6 to 9 and hence had at least a moderate quality (Supplementary Appendix 2). For the two randomized controlled trials included, the risk of bias assessment results are summarized in Figure 2.

### Prevalence of cryptococcal meningitis among people living with HIV

Of the 17 studies included, 9 reported on the prevalence of CM among PLWH (Table 1).

**TABLE 1:** Characteristics of studies included in the prevalence meta-analysis of CM among PLWH in Africa, 1995–2021.

Author	Publication year	Country	Sample size	Cases	Prevalence 95% CI (%)
Deiss et al. (38)	2021	Mozambique	1,795	96	5.35
Letang et al. (39)	2015	Tanzania	750	11	1.47
Bergemann and Karstaedt (40)	1996	South Africa	284	37	13.03
Soumare et al. (41)	2005	Senegal	45	9	20.00
Bamba et al. (42)	2012	Burkina Faso	5,129	61	1.19
Apetse et al. (43)	2011	Togo	1,764	51	2.89
Oumar et al. (44)	2008	Mali	204	17	8.33
Luma et al. (37)	2013	Cameroon	672	75	11.16
Lakoh et al. (45)	2020	Sierra Leone	170	5	2.94

CI, Confidence interval.

**FIGURE 3:**
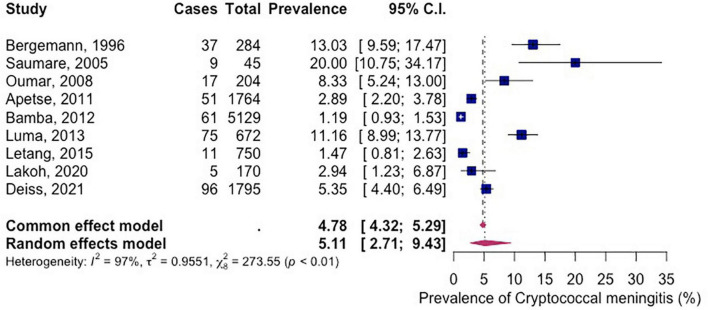
Forest plot of prevalence of HIV related cryptococcal meningitis in Africa (random effects), 1995–2021.

The reported prevalence ranged from 1.2 to 20.00%. The prevalence of CM in PLWH in Africa was 5.11% (95% CI 2.71–9.43%; participants = 10,813; studies = 9; *I*^2^ = 97%) (Figure 3). There was high heterogeneity among included studies (*P* < 0.01).

**FIGURE 4:**
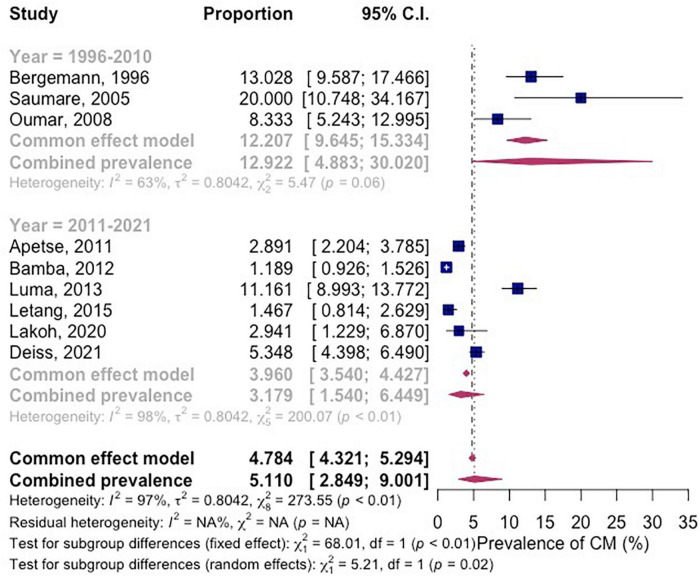
Forest plot of subgroup analysis by year of the studies were conducted for pooled prevalence in included studies.

The subgroup analysis shows that the prevalence of CM among PLWH between the years 1996–2010 was 12.9% (95% CI 4.883–30.0; participants = 533; studies = 3; *I*^2^ = 63%) and between the years 2011–2021 was 3.18% (95% CI 1.54–6.45; participants = 10,280; studies = 6; *I*^2^ = 98%) (Figure 4). There is significant subgroup difference between the two groups (*P* = 0.02).

The meta-regression result shows that 96.59% of the observed variance about the regression line reflects variation in true effects rather than sampling error. The test for heterogeneity yields a Q-value of 205.53 with 7 degrees of freedom and a corresponding *p*-value of < 0.0001. We concluded that the model fully explains the variation in effects. The *R*^2^ analog is 15.80%, which tells us that the model can explain some 16% of the variance in prevalence. The coefficient was –1.5083 (95% CI –2.28 to 0.21). Between the years 2011–2021, the prevalence of CM in Africa decreased by 51% as compared to the year 1996–2010 (Table 2).

**TABLE 2:** Meta-regression.

	Estimate	SE	*Z*-value	*P*-value	CI.lb	CI.ub
Intercept	–1.9106	0.4906	–3.8946	0.00001	–2.8722	–0.9491
Year 2011–2021	–1.4913	0.5831	–2.5577	0.0105	–2.6341	–0.3485

**FIGURE 5:**
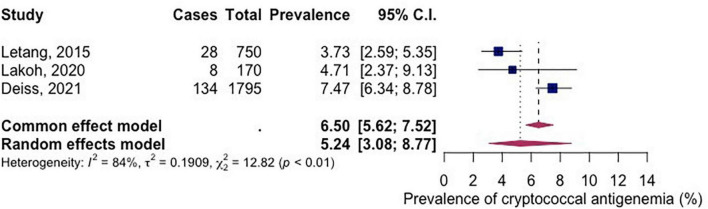
Forest plot of studies included which also reported on cryptococcal antigenemia.

Three studies had reported the prevalence of cryptococcal antigenemia. The pooled prevalence of cryptococcal antigenemia (among included studies) in PLWH in Africa between the year 2015–2021 was 5.24% (95% CI 3.08-8.77; participants = 2,715; studies = 3; *I*^2^ = 84%) (Figure 5).

**FIGURE 6:**
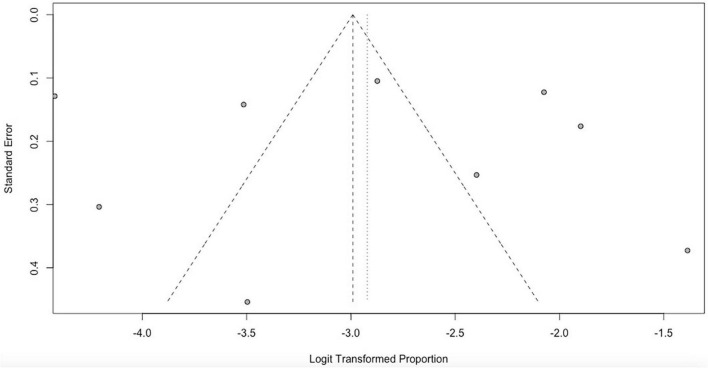
Funnel plot to assess publication bias for pooled prevalence (random effects).

The funnel plot showed that all studies lay symmetrically around the pooled effect estimate implying that there was no publication bias (Figure 6).

### Predictors of mortality

Of the 17 studies included, 9 reported the predictors of mortality among PLWH on CM induction therapy (Table 3). Majority of participants were male aged 32–36 years, with all studies reporting CD4 counts less than 100 cells/ml of which, eight of nine studies reported CD4 less than 50 cells/ml. Four studies reported some history of prior ART use among participants while only two studies mentioned that all participants were ART naïve. Furthermore, majority of the participants were treated with one or a combination of Amphotericin, Fluconazole, and Flucytosine.

**TABLE 3:** Characteristics of included studies in the narrative synthesis of mortality predictors among PLWH on induction therapy in Africa, 1998–2018.

Author	Year	Country	Sample size	Study design	% Men	Median/mean age	Median CD4 cells/mm^3^	ART history	CM diagnosis confirmed by	Treatment	% death
Luma et al. (37)	2013	Cameroon	672	CS	57.3	36.9	23	No data	India Ink/CT	Fluconazole	5.8
Patel et al. (4)	2018	Botswana	236	C	69	36	39	57%	India Ink/CSF CRAG/CSF Culture	Amphotericin, Fluconazole	67.3
Nussbaum et al. (46)	2010	Malawi	41	RCT	66	36	21	All naïve	India Ink/CSF CRAG/CSF Culture	Fluconazole, Flucytosine	48.8
Kizza et al. (47)	1998	Uganda	58	RCT	47	34	77	No data	India Ink/CSF CRAG/SCF Culture	Fluconazole, Flucytosine	46.6
Longley et al. (48)	2008	Uganda	60	C	57	34	12	All naïve	India Ink/CSF Culture	Fluconazole	51.7
Lightowler et al. (49)	2010	South Africa	186	CSe	49.5	32	36	14.5%	CSF Culture/India ink	Amphotericin, Fluconazole	33.9
Gaskell et al. (50)	2014	Malawi	58	C	51	35	36	45%	India Ink. CSF Culture	Fluconazole	44.9
Hiesgen et al. (51)	2017	South Africa	87	C	55.2	34	52	44.8%	India Ink/CSF CRAG/CSF Culture	Amphotericin, Fluconazole	31
Bicanic et al. (52)	2009	Thailand, South Africa, Uganda	262	C	45	33	26	No data	CSF Culture	Amphotericin, Fluconazole, Flucytosine	31

CSF, cerebral spinal fluid; CRAG, Cryptococcal antigen; ART, antiretroviral therapy; CD4, cluster of differentiation 4; CM, cryptococcal meningitis; CT, computerized tomography scan; CSF, cerebral spinal fluid;%, percentage; CS, Cross-sectional; C, Cohort; RCT, Randomized control trial; CSe, Case series.

At least two studies reported FLU monotherapy, focal neurological signs, low GCS and delayed diagnosis of CM as predictors of mortality regardless of time point. FLU monotherapy was reported by at least two studies while focal neurological symptoms, DBP < 60 mmHg, and current TB coinfection (TB) were reported as significant predictors of mortality by a single study at both 2 and 10 weeks timepoints. Other significant predictors reported by at least one study at 2 weeks timepoint includes prior ART use and high serum sodium level. Additionally, low CD4 count, altered level of consciousness, high white blood cell count, low CSF white cell count and high serum sodium were reported as predictors of mortality at 10 weeks timepoint (Table 4).

**TABLE 4:** Summary of reported predictors of mortality for HIV-related CM for each study included in the narrative synthesis.

Authors	Overall mortality predictors reported in a study	Reported predictors at 2 weeks	Reported predictors at 10 weeks
Luma et al. (37)	Male sex, younger 33.5 median, headache, neck stiffness, focal signs, cerebral edema on CT, Low CD4	No data	No data
Patel et al. (4)	Low GCS, older > 50 years, aneamia, high WBC, low CSF WCC, renal dys, low sodium,	High WBC, low CSF WCC, No Amp B (induction)	ART hx, high sodium, No Amp B
Nussbaum et al. (46)	Flu monotherapy	Flu monotherapy	Flu monotherapy
Kizza et al. (47)	Diagnosis delay, lack of supportive care	No data	No data
Longley et al. (48)	Diagnosis delay, Low GCS	No data	No data
Lightowler et al. (49)	Focal neurological symptoms, DBP < 60, current TB, Flu monotherapy	Focal neurological symptoms, DBP < 60, current TB, Flu monotherapy	Focal neurological symptoms, DBP < 60, current TB, Flu monotherapy, missing CD4
Gaskel et al. (50)	Fluconazole monotherapy	No data	No data
Hiesgen et al. (51)	ART naïve, ART default, N&V history	No data	No data
Bicanic et al. (52)	Low GCS, high fungal load, slow fungal clearance	No data	No data

TB, tuberculosis; GCS, Glasgow coma scale; AmB, Amphotericin B; CT, computerized tomography scan; N&V, nausea and vomiting; ART, Antiretroviral therapy; DBP, diastolic blood pressure; WCC, CSF white cell count; WBC, white blood cells; No data- no data on predictors reported for the time point.

## Discussion

CM is an important cause of adult meningitis in PLWH presenting with severe immunosuppression (3). Though ART access has improved *via* the WHO test and treat program, CM continues to be perpetuated by failures in the ART systems apparent in Africa (2). Our systematic review and meta-analysis of 12,473 participants aimed to establish the prevalence of CM in HIV-positive individuals and its predictors of mortality.

The majority of the studies in our review confirmed CM diagnosis by India ink/CSF culture. CSF fungal culture is the gold standard and is often preferred over Indian ink for its higher sensitivity (53). However, culture has drawbacks which include being more expensive and delaying diagnosis by 3–7 days (54). The use of serum cryptococcal antigen (CRAG) testing is indicated as a screening method for likely CM in advanced HIV and is only used to commence prophylactic therapy among CRAG-positive individuals (14, 55).

In this review, over 80% of the studies included reported a CD4 count of less than 50 cells/mm^3^ at baseline. Unlike most studies that suggest that the occurrence of CM is common in those with CD4 count less than 200, our study found an even lower CD4 count as a common finding (23, 56). This could explain why the majority of our studies had a mortality percentage greater than 30% which was comparable to 44% (short-term mortality) reported by Tenforde et al. (31) and 36.7% by Majumder et al. (57). As the majority of the included studies reported less than 50% prior ART use or none at all, we infer that our population had advanced immunosuppression and hence explaining the much lower than usual CD4 count. Both low CD4 count and delayed ART initiation are risks for opportunistic infections such as CM among PLWH.

Our review results showed that the prevalence of CM among PLWH in Africa ranged between 1.4 and 20.0% with a pooled prevalence of 5.11% (95% CI 2.71–9.43%; participants = 10,813; studies = 9; *I*^2^ = 97%). This finding was not similar to the global percentage of CM in AIDS-related deaths which stands at 15% (3). The result was comparable to a global burden of disease study from 2009 which estimated the burden of CM among PLWH between 0.04 and 12% with SSA leading in annual numbers (58). In contrast, our result was much lower than that from a western Indian study that reported a 10-year prevalence ranging between 49 and 100%. In that study, only 23.3% of its participants were HIV positive and just over half of them tested positive for CM (59). This implies that the overall CM prevalence among PLWH in the study could have been much lower or even closer to our African estimate. A systematic review by Derbie et al. found the prevalence of cryptococcal antigenemia to be 8% (95% CI 6–10) which was almost double our prevalence for CM which was logical since not all cases of cryptococcal antigenemia result in invasive cryptococcal disease (55). Although cryptococcal antigenemia is not equivalent to a diagnosis of CM, it has been thought of as an important proxy measure for CM in PLWH (60).

Meta-regression of prevalence comparing the period from 1996 to 2010 and that from 2011 to 2021 demonstrated a significant reduction of HIV-related CM of about 51%. This observation could be credited to the evolved WHO ART treatment guideline that included triple therapy for all HIV-positive patients regardless of CD4 count (61). Early ART initiation prevents the occurrence of opportunistic infections (62).

Biases may have been introduced in our calculation of pooled prevalence since included studies only focused on patients who presented to the health facility. This may have introduced participant selection bias and undermined our disease prevalence. Additionally, patient populations from the studies included had inert variations that could have influenced our synthesized prevalence. Our prevalence conclusion may have been overestimated, as our study utilized data for PLWH with CD4 counts less than 100 cells/ml which could be more representative of an annual incidence for persons with low CD4 count in Africa.

This study found that low GCS (< 15/15), focal neurological signs, delay in CM diagnosis, and FLU monotherapy were significant predictors of mortality for CM in PLWH. Low GCS, defined as a score < 15/15 or a reduced level of consciousness, was supported as a mortality predictor by studies looking at both short-term and long-term mortality predictors which applied cox regression models to establish associations (57, 63). Another study done in Uganda linked low GCS to seizure occurrence and these were associated with higher 10-week mortality (64). Delayed CM diagnosis results in an increase of fungal load which in turn accelerates the occurrence of raised intracranial pressures causing a lower GCS and focal neurological symptoms (65–67). Interventions such as serial CSF drainage *via* lumbar puncture in addition to antifungal therapy, continue to prove invaluable to patient survival (68). In 2018, WHO introduced a strategy to reduce death from HIV-related CM with the use of combined antifungal therapy and routine serum CRAG screening. Our study found that FLU monotherapy, though not a standard of care regimen, had continued to be majorly used in Africa and this was according to Loyse et al. (69). This was possibly perpetuated by the high cost, need for in-hospital administration and need for frequent laboratory monitoring of AmB and 5FC (61, 70). FLU monotherapy even at high doses continues to be a sub-optimal treatment option for HIV-related CM due to its fungistatic nature that results in a slow fungal clearance rate and consequently a higher 2 and 10-week mortality rate (54, 71, 72).

Possible predictors for mortality at 2 weeks were found to be low CSF white cell count (WCC), focal neurological symptoms and FLU monotherapy. Low CSF white cell count was also reported as a mortality predictor at day-14 by a study done in Thailand and United States (73). Patients diagnosed with CM in HIV usually have a CD4 count < 200 cells/mm^3^ which translates to an inability to mount an adequate immune response, which could explain why low WCC was found to be a significant risk factor for early mortality (74). Focal neurological signs in CM occur as a result of raised intracranial pressure or the presence of space-occupying lesions. A study from Thailand documented focal deficits in 12% of participants while an Indian study found it in 13% of their participants (70, 75). Only 50% of our studies reported a raised intracranial pressure while others mentioned that measuring this was not possible due to lack of equipment or that it was not routinely done. Delayed fungal clearance that is associated with monotherapy could explain why FLU monotherapy was significant at as early as 2 weeks of therapy.

In this study, FLU monotherapy was found to be the only risk factor reported by at least 2 of the included articles as a predictor of mortality at 10 weeks. This finding consolidates evidence that though an inferior treatment option, FLU monotherapy remains largely used in Africa due to the high cost of AmB and unavailability of 5FC (69).

Interestingly history of ART use was indicated by one article as a predictor of mortality at 10 weeks. It is well known that the timing of ART in those found to have CM is key, due to the risk of immune reconstitution syndrome (IRIS). It is therefore logical to expect that those already on ART before CM diagnosis would have a higher CD4 count and hence better outcomes (14). Our result though seemingly contradictory, was similar to results from a study done by the Caribbean, Central and Southern American Network for HIV Epidemiology. They found that among 340 adult patients with HIV, mortality was high among those that had started ART 2–3 years before CM diagnosis (*p*-value = 0.03) (76). This may be due to the presence of virological failure (secondary to poor adherence) coupled with immunological failure resulting in CM infection. It may also have been due to a reactivation of “dormant” prior CM infection or IRIS (77, 78). Another study done in Uganda stratified 605 participants as ART naïve or not and also by duration on ART before CM diagnosis. They found no difference in the 2-week mortality by ART groups but those who received ART ≤ 14 days prior to CM diagnosis had higher mortality of about 47%. This agrees with our study that found no association with ART history at 2 weeks.

Using retrospective studies to predict the outcome of HIV-related CM is difficult due to variabilities in patient’s treatment regimens, routine care such as serial LP and comorbidities. Further, not all studies used regression and survival analysis in addition to the linear correlation between factors assessed. The result was inadequate accounting for possible overlapping effects that may have confounded and overestimated the strength of associations.

This systematic review only included studies that were published or translated into the English language, and this may have resulted in the exclusion of possibly relevant studies, hence reducing our precision. Included studies did not have a standardized data collection method with relation to outcome measurements and their time points. This affected our capacity to conclude using more inferential statistical methods rather than simple narrative analysis. We were also unable to quantitatively estimate the excess risk of identified risk factors which unadvertently decreased statistical power or usefulness of our conclusion on mortality risk. Sparse data were available for inclusion in our review as the majority of studies undertaken in Africa looked at cryptococcal antigenemia rather than CM prevalence. Cryptococcal antigenemia is not a proxy for CM prevalence as they are very different disease states, but it remains useful for generating data on CM screening and early treatment policies. Our study was, however, one of the first to attempt to systematically review the pooled prevalence of CM for PLWH in Africa and its mortality predictors.

## Conclusion

The findings indicate that CM remains of concern in Africa despite the increase in ART coverage among PLWH. The subgroup analysis shows that the prevalence of CM has significantly decreased from 1996–2010 to 2011–2021 among PLWH on induction therapy living in Africa. FLU monotherapy, focal neurological symptoms, diastolic blood pressure (DBP) < 60 mmHg, and current tuberculosis coinfection were significant predictors of mortality at 2- and 10-weeks time points. Efforts must be made to increase the availability of standard antifungal therapy for implementing early combined induction therapy in Africa.

## Data availability statement

The original contributions presented in this study are included in the article/Supplementary material, further inquiries can be directed to the corresponding author/s.

## Author contributions

SM and DA developed the protocol, reviewed the reference list, extracted data, and entered it into R-studio for analysis, performed the analysis, constructed the summary tables, and extracted result figures. SM, DA, CP, TG, SS, GY, and TM were responsible for ensuring quality of the data reported and manuscript review. All authors have read and approved the final manuscript.

## Abbreviations

ACTA: Advancing Cryptococcal Meningitis Treatment for Africa
AIDS: Acquired Immuno-Deficiency Virus
AmB: Amphotericin B
ART: Antiretroviral Therapy
ATT: Anti-Tuberculous Therapy
CD4: cluster of differentiation 4
CM: Cryptococcal Meningitis
CRAG: Cryptococcal Antigen
CSF: Cerebral Spinal Fluid
CT: computerized tomography scan
DBP: Diastolic blood pressure
GCS: Glasgow coma scale
5FU: Flucytosine
FLU: Fluconazole
Hgb: Hemoglobin
HIV: Human Immuno-deficiency Virus
ICP: Intracranial Pressure
LMIC: Low and Middle-Income Countries
LP: Lumbar Puncture
PEP: Post Exposure Prophylaxis
PICO: Population intervention, comparison and outcome
PLWH: People Living With HIV
PrEP: Pre-exposure Prophylaxis
PRISMA: Preferred Reporting Items for Systematic Reviews and Meta-Analyses
PTB: Pulmonary Tuberculosis
RCT: Randomized controlled trial
SBP: Systolic blood pressure
SSA: Sub-Saharan Africa
TB: Tuberculosis
WBC: White blood cells
WCC: White cell count
IDSA: Infectious Disease Society of America.
