# Supplementary material for: Prevalence of cryptococcal meningitis among people living with human immuno-deficiency virus and predictors of mortality in adults on induction therapy in Africa: A systematic review and meta-analysis
Source: Front Med (Lausanne). 2022 Sep 8;9:989265. doi: 10.3389/fmed.2022.989265 (PMC9494297; doi:10.3389/fmed.2022.989265)
Supplement: Supplementary file 1 [file Data_Sheet_1.PDF]

SUPPLEMENTARY MATERIAL

Appendix 1: Search plan

PubMed (25 May 2021)	#2<br>Search: "meningitis, cryptococcal"[MeSH Terms] OR "cryptococcal"[Text Word] OR "cryptococcosis"[Text Word] OR "Cryptococcus neoformans"[Text Word] OR "cryptococcus gattii"[Text Word] OR "cryptococc*"[Text Word] AND (1990:3000/12/12[pdat]) Filters: from 1990 - 3000/12/12<br>#3<br>Search: "HIV"[MeSH Terms] OR "human immunodeficiency virus"[Text Word] OR "AIDS"[Text Word] Filters: from 1990 - 3000/12/12<br>#4<br>Search: "Death"[MeSH Terms] OR "Mortality"[MeSH Terms] OR "Mortality"[MeSH Subheading] OR "death*"[Text Word] OR "died"[Text Word] OR "non survivor*"[Text Word] OR "non survival"[All Fields] OR "poor outcome*"[Text Word] OR "fatal"[Text Word] Filters: from 1990 - 3000/12/12<br>#5<br>Search: "africa"[MeSH Terms] OR africa[Text Word] Filters: from 1990 - 3000/12/12<br><br>#6 (COMBINED SEARCH=172 HITS)<br>Search: ((("meningitis, cryptococcal"[MeSH Terms] OR "cryptococcal"[Text Word] OR "cryptococcosis"[Text Word] OR "Cryptococcus neoformans"[Text Word] OR "cryptococcus gattii"[Text Word] OR "cryptococc*"[Text Word] AND (1990:3000/12/12[pdat]) AND (1990:3000/12/12[pdat])) AND ("HIV"[MeSH Terms] OR "human immunodeficiency virus"[Text Word] OR "AIDS"[Text Word] AND (1990:3000/12/12[pdat]))) AND ("Death"[MeSH Terms] OR "Mortality"[MeSH Terms] OR

	"Mortality"[MeSH Subheading] OR "death*"[Text Word] OR "died"[Text Word] OR "non survivor*"[Text Word] OR "non survival"[All Fields] OR "poor outcome*"[Text Word] OR "fatal"[Text Word] AND (1990:3000/12/12[pdat]))) AND ("africa"[MeSH Terms] OR africa[Text Word] AND (1990:3000/12/12[pdat])) Filters: from 1990 - 3000/12/12<br><br>#9 (COMBINED with prevalence and risk =5 hits)<br><br>Search: ((((("meningitis, cryptococcal"[MeSH Terms] OR "cryptococcal"[Text Word] OR "cryptococcosis"[Text Word] OR "Cryptococcus neoformans"[Text Word] OR "cryptococcus gattii"[Text Word] OR "cryptococc*"[Text Word] AND (1990:3000/12/12[pdat]) AND (1990:3000/12/12[pdat])) AND ("HIV"[MeSH Terms] OR "human immunodeficiency virus"[Text Word] OR "AIDS"[Text Word] AND (1990:3000/12/12[pdat]))) AND ("Death"[MeSH Terms] OR "Mortality"[MeSH Terms] OR "Mortality"[MeSH Subheading] OR "death*"[Text Word] OR "died"[Text Word] OR "non survivor*"[Text Word] OR "non survival"[All Fields] OR "poor outcome*"[Text Word] OR "fatal"[Text Word] AND (1990:3000/12/12[pdat]))) AND ("africa"[MeSH Terms] OR africa[Text Word] AND (1990:3000/12/12[pdat])) AND (1990:3000/12/12[pdat])) AND ("Risk Factors"[Mesh] AND (1990:3000/12/12[pdat]))) AND ("Prevalence"[Mesh] AND (1990:3000/12/12[pdat])) Filters: from 1990 - 3000/12/12
EMBASE (28th May 2021)	#1 AND #2 AND #3 AND #4 AND #6<br>91 hits -------6 included (final)<br>#8<br>#1 AND #2 AND #3 AND #6<br>160 hits<br>#7

	#1 AND #2 AND #3 AND #4 AND #5 AND #6<br>7 hits<br>#6<br>'africa'<br>341,108 hits<br>#5<br>'risk factor'<br>1,251,524 hits<br>#4<br>'mortality'<br>1,629,012 hits<br>#3<br>'prevalence'<br>1,147,556 hits<br>#2<br>('human immunodeficiency virus' OR 'human immunodeficiency virus infection' OR 'human immunodeficiency virus infected patient') AND ([embase]/lim OR [medline]/lim OR [pubmed-not-medline]/lim)<br>470,429<br>#1<br>('cryptococcal meningitis'/exp OR 'cryptococcal meningitis' OR 'cryptococcosis'/exp OR 'cryptococcosis') AND ([embase]/lim OR [medline]/lim OR [pubmed-not-medline]/lim)
Google Scholar [25 May 2021]	"Cryptococcal meningitis" AND "Human Immunodeficiency virus" OR PLWH AND Mortality OR Death AND Risk factors OR determinants AND prevalence AND Africa<br><br>First 10 pages = 100 [10 per page]
MedLine (via Hinari -Pubmed and embase)	

Appendix 2: Assessing the risk of bias in included studies

a) Adapted Newcastle Ottawa Risk of Bias tool for observational studies included in the prevalence meta-analysis

Study	Selection(1)				Comparability(2)	Outcome(3)		Score	Quality
	Representativeness: Truly(all/random)*, somewhat avaerage pop (non random)*, Selected/no description	Sample size; Justified/ Satisfactory*, Not	Responders vs Non responders described*, Poorly, Not	Risk factor ascertained; Validated*, Non validated*, None	Subjects are comparable, confounding factors controlled; Most*, Additional*	Assessment; Independent Blind**, Record linked**, Self reported*, No description	Statistical test; Clear & appropriate*, Not	Good >/= 8, Moderate 6- 7, Poor</= 5	
Deiss 2021	*	*		*	*	**	*	7	**moderate**
Lakoh 2020	*	*		*	*	**	*	7	**moderate**
Letang 2015	*	*	*	*	**	**	*	9	**good**
Luma 2013	*	*		*		**	*	6	**moderate**
Bamba 2012	*	*		*	*	**		6	**moderate**
Apetse 2011	*	*	*	*	*	**		7	**moderate**
Oumar 2008	*	*		*	*	**	*	7	**moderate**
Soumare 2005	*	*		*	*	**		6	**moderate**
Bergemann 1996	*	*	*		*	**		6	**moderate**

b) Adapted Newcastle Ottawa Risk of Bias tool for observational studies included in the narrative synthesis

Study	Selection (1)				Comparability (2)	Outcome(3)		Score	Quality
	Representativeness; Truly(all/random)*, Somewhat average pop(non-random)*, selected/no description	Sample size: Justified/ satisfactory*, Not	Responders vs Non resp. described*, Poorly, Not	Risk factor ascertained : Validated*, Non validated*, None	subjects are comparable, confounding factors controlled; Most*, Additional*	Assessment- Independent blind**, Record linked **, Self report*, No description	Stat. test Clear&appropraite *, not	Good >/= 8, moderate 6-7, Poor </= 5	
Luma	*	*		*	*	**	*	7	moderate
Patel	*	*		*	*	**	*	7	moderate
Longley	*		*	*	**	**	*	8	good
Lightowler	N/A								N/A
Gaskel	*			*	*	**	*	6	moderate
Hiesgen	*	*		*	*	**	*	7	moderate
Bicanic T, :	*			*	*	**	*	6	moderate

c) Cochrane collaborations tool for assessing risk of bias

QUALITY ASSESSMENT OF RCTS

	**Risk of bias**	**Author judgement**
**1.Mayanga 1998**		
Random sequence generation (selection bias)	Low risk	Sequence generation not described but randomisation described using sealed envelopes
Allocation concealment (selection bias)	Low risk	Randomisation allocation to group
Blinding of participants and personnel (performance bias)	Unclear	Not described
Blinding of outcome assessment (detection bias)	Unclear	Not described
Incomplete outcome data (attrition bias)	Low risk	Data for all patients was recorded
Selective reporting (reporting bias)	Low risk	All pre-specified outcomes were reported
Other bias	High risk	Grant from pharmaceutical company
**2.Nussbaum 2010**		
Random sequence generation (selection bias)	Low risk	Computer generate sequence
Allocation concealment (selection bias)	Low risk	Random sequence allocation
Blinding of participants and personnel (performance bias)	Unclear	Not clearly mentioned
Blinding of outcome assessment (detection bias)	High risk	Not blinded
Incomplete outcome data (attrition bias)	Low risk	Data on all participants reported
Selective reporting (reporting bias)	Low risk	All outcomes reported on
Other bias	unclear	
